# Temperature Monitoring in Hyperthermia Treatments of Bone Tumors: State-of-the-Art and Future Challenges

**DOI:** 10.3390/s21165470

**Published:** 2021-08-13

**Authors:** Francesca De Tommasi, Carlo Massaroni, Rosario Francesco Grasso, Massimiliano Carassiti, Emiliano Schena

**Affiliations:** 1 Unit of Measurements and Biomedical Instrumentations, Department of Engineering, Università Campus Bio-Medico di Roma, Via Alvaro del Portillo, 00128 Rome, Italy; f.detommasi@unicampus.it (F.D.T.); c.massaroni@unicampus.it (C.M.); 2 Unit of Interventional Radiology, School of Medicine, Università Campus Bio-Medico di Roma, Via Alvaro del Portillo, 00128 Rome, Italy; r.grasso@unicampus.it; 3 Unit of Anesthesia, Intensive Care and Pain Management, School of Medicine, Università Campus Bio-Medico di Roma, Via Alvaro del Portillo, 00128 Rome, Italy; m.carassiti@unicampus.it

**Keywords:** bone tumors, CT thermometry, fiber Bragg grating sensors, fluoroptic sensors, hyperthermia treatments, MR thermometry, thermocouples, thermistors, temperature monitoring, ultrasound thermometry

## Abstract

Bone metastases and osteoid osteoma (OO) have a high incidence in patients facing primary lesions in many organs. Radiotherapy has long been the standard choice for these patients, performed as stand-alone or in conjunction with surgery. However, the needs of these patients have never been fully met, especially in the ones with low life expectancy, where treatments devoted to pain reduction are pivotal. New techniques as hyperthermia treatments (HTs) are emerging to reduce the associated pain of bone metastases and OO. Temperature monitoring during HTs may significantly improve the clinical outcomes since the amount of thermal injury depends on the tissue temperature and the exposure time. This is particularly relevant in bone tumors due to the adjacent vulnerable structures (e.g., spinal cord and nerve roots). In this Review, we focus on the potential of temperature monitoring on HT of bone cancer. Preclinical and clinical studies have been proposed and are underway to investigate the use of different thermometric techniques in this scenario. We review these studies, the principle of work of the thermometric techniques used in HTs, their strengths, weaknesses, and pitfalls, as well as the strategies and the potential of improving the HTs outcomes.

## 1. Introduction

Cancer is one of the leading causes of mortality worldwide, with an ever-growing number of people affected. By 2025, it is estimated over 20 million people will suffer from this disease each year [1]. In this context, patients facing primary lesions in the breast, lung, and prostate have a propensity to inherit bone metastases during their illness, with an estimated incidence of more than 60% [2,3,4,5,6]. In addition to metastatic lesions, osteoid osteoma (hereafter OO) is a quite frequent benign tumor involving bone with small dimensions (up to 1.5 cm in diameter), affecting mainly young patients [7,8]. Whereas bone metastases primarily have spine involvement [9,10], OO is predominantly in anatomical sites such as the femur and tibia [8,11]. Patients affected by bone lesions (both primary and metastatic ones) suffer from acute pain intensifying during the night, thus resulting in a poor-quality life [12,13]. In this scenario, pain reduction in a lasting way is a priority, especially in patients with low life expectancy [14]. To date, a standard treatment for managing such kinds of lesions is radiotherapy, as stand-alone dealing or in conjunction with surgery, which remains the gold standard, especially in patients with bone fractures involvement [15,16,17]. Nevertheless, both these approaches exhibit some limitations: radiotherapy may need some weeks before providing possible benefits, low radiation tolerance for tumors in the proximity of vulnerable structures, and inability to treat patients with previous comorbidities [14,18].

For almost thirty years, hyperthermia treatments (hereafter HTs) have been gaining momentum in this arena. Such procedures have emerged as valuable alternatives to traditional therapies in bone cancer, owing to their minimally invasive nature [19,20,21,22]. Given the success rate in tumor control and the immediate effect of pain relief, HTs have been identified as a possible treatment in bone metastases by the National Comprehensive Cancer Network in its November 2020 guidelines [23]. The main principle of HTs is to achieve complete and effective cancer removal by raising cytotoxic temperatures (i.e., >50 °C) [24]. Among HTs, radiofrequency ablation (RFA), laser ablation (LA), and microwave ablation (MWA) are well documented in the literature for bone malignancy management [11,25,26,27,28,29]. Such procedures are performed via percutaneous access whereby a needlelike applicator is positioned within the tumor tissue under imaging guidance (e.g., computed tomography -CT-, magnetic resonance -MR-) [30,31]. RFA, LA, and MWA working principles differ according to the energy source employed, and cell necrosis is achieved by a localized increase in temperature because of energy-tissue interaction [24]. Differently from RFA, LA and MWA, high intensity focused ultrasound (hereafter HIFU) represents another technique belonging to the hyperthermia-based procedures totally non-invasive since it does not require the insertion of a needlelike applicator inside the treated area. In HIFU, the temperature rising is accomplished by means of a mechanical transducer placed on the external body surface corresponding to the area to be treated [32]. In the last decades, HIFU has been broadly exploited to treat bone tumors owing to the promising results reported, especially in terms of pain relief [33,34,35,36].

During HTs, the amount of thermal injury is strongly related to the temperature experienced by the tissue during the procedure and the exposure time, as outlined by the most popular models (e.g., Arrhenius’ law, CEM 43 °C [37]). Temperature monitoring in HTs is paramount to ensure damage to the tumor portion plus a reasonable safety margin while preserving healthy surrounding anatomical structures [38,39]. Therefore, keeping track of temperature changes over time accounts for valuable information to the clinician performing the procedure. Real-time temperature understanding allows adjusting treatment settings (e.g., input power and treatment time) to clearly identify the endpoint and ensure the safety of the procedure [40,41,42]. To date, commercially available hyperthermia systems are equipped with temperature sensors embedded within the energy source. However, this solution does not provide information about heat propagation inside the tissue undergoing ablation while exclusively intended to ensure temperatures at the source tip not exceeding a safety value generally set on the HT systems. Otherwise, temperature tissue monitoring may be accomplished by many either contact or contactless techniques with different purposes. Among others, temperature map reconstruction resulting from tissue temperature measurements allows accurately estimating tissue damage, thus achieving a good match between the portion of tissue that should be damaged and the one that experiences cytotoxic temperatures during the procedure. Temperature knowledge gains further relevance in bone tumors growing adjacent to vulnerable structures such as the spinal cord and nerve roots [43]. The dealing of such lesions is characterized by the major challenge of preventing cytotoxic temperatures in susceptible areas [44]. Indeed, neural elements are not allowed experiencing temperatures higher than 45 °C since this would lead to permanent damage including in the worst cases paralysis or paresis that severely impact the patients’ status [22,45,46,47]. Therefore, both thermal insulation techniques and temperature monitoring are mandatory in this scenario to improve the procedure’s safety and efficacy [46,48,49]. While the firsts are carried out by injecting CO_2_ or saline solution to create thermal dissection, temperature monitoring leads the way to correctly gather temperature information in the treated tissue without compromising sensitive elements [46,50,51]. Moreover, temperature measurement plays a key role in investigating the effectiveness of either available or novel ablation devices, thus affording optimization and understanding their performances, as well as gaining new findings on how various HT settings affect the treatment effects. In addition, single-point temperature measurements are broadly accepted in clinical settings to protect vulnerable anatomical areas from cytotoxic temperatures. In these cases, thermometers must be carefully inserted in the proximity of these structures by avoiding undesirable injuries.

In spite of this potential impact, temperature monitoring is not well established in clinical settings since it presents several open challenges when performed during HTs. 

In this paper, we will provide an overview of the principal thermometric techniques used in the field of HTs, focusing on the specific application in bone cancer. The widespread solutions will be presented by briefly describing their working principle and the principal strengths and weaknesses of using such technologies in this scenario. Next, we will describe state-of-the-art related to these thermometric techniques in each bone HT (i.e., RFA, LA, MWA, and HIFU). Finally, we will emphasize our personal point of view concerning the current status with the aim to provide valuable insights, current and future challenges on this topic.

## 2. Temperature Monitoring: Main Techniques and Applications in Bone HTs

Extensive investigations have been devoted to providing suitable solutions for continuous temperature monitoring during HT since the knowledge of temperature may be beneficial to ensure the procedure’s safety.

This section is devoted to describing the most popular techniques used for temperature monitoring in HTs of bone cancer in preclinical and clinical studies.

Firstly, the principle of work of the most popular thermometric techniques is described, along with the main strengths, weaknesses, and pitfalls. Then, applications of the mentioned techniques in HTs of bones will be described: Section 2.2 will be devoted to in-bone temperature monitoring during RFA, Section 2.3 during LA, Section 2.4 during MWA, and Section 2.5 during HIFU. 

### 2.1. Most Popular Thermometric Techniques Used during HTs

**Figure 1:**
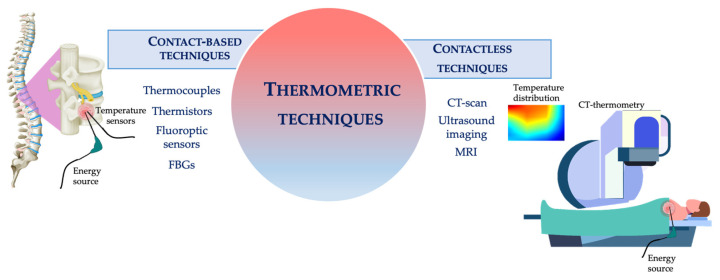
Schematic representation of the thermometric techniques employed during HTs.

Basically, thermometric techniques employed in this scenario can be classified as either contact-based or contactless methods (see Figure 1) [39]. Contact-based techniques involve the insertion of the sensing element within the treated tissue. This category includes thermocouples, thermistors, fluoroptic, and fiber Bragg grating (FBG) sensors. Contactless techniques do not require direct contact with the measurement site. This category involves diagnostic imaging techniques (i.e., magnetic resonance imaging, MRI, computed tomography, CT, and ultrasound thermometry) [39].

#### 2.1.1. Contact Based Techniques

A thermocouple consists of two different metal wires (conductors A and B) joined together in correspondence of the so-called hot junction. This is the junction where the temperature to be measured is applied. The other extremity (i.e., the cold junction) consists of two free conductors at known temperature. Thermocouple’s working principle is based on Seeback effect. When a temperature gradient between the two junctions occurs, an electromotive force (emf) can be measured, as evidenced by the equation [52]:

(1) $$\mathrm{emf}=\int_{T_{\mathrm{ref}}}^{T_{1}}S_{A}\left(T\right)\;\mathrm{dT}-\int_{T_{\mathrm{ref}}}^{T_{1}}S_{B}\;\mathrm{dT}=\int_{T_{\mathrm{ref}}}^{T_{1}}S_{\mathrm{AB}}\left(T\right)\;\mathrm{dT}$$

where T_ref_ and T_1_ represent the cold junction temperature and the temperature to be measured, respectively. S_A_, S_B_, and S_AB_ denote the Seeback coefficients for conductor A, B and joined conductors (i.e., thermocouple), respectively. According to the applications, it is possible to choose a specific type of thermocouple by means of a different conductors’ combination (e.g., chromel/alumel, copper/constantan, iron/constantan). Each combination is characterized by different performances in terms of sensitivity, accuracy, and measurement range. Thermocouples found application in HTs for the first time in 1935 [53]. After this early investigation, several studies have evidenced their use during HTs [54,55,56,57,58,59,60]. The widespread acceptance of thermocouples is attributable primarily to their low cost, small size, robustness, wide measuring range and fast response time [61]. Nevertheless, the main drawback is related to the metallic composition not allowing their use in the presence of high electromagnetic fields (e.g., during MR scans). In case of LA, the metallic components may cause a measurement artifact due to the absorption of light [62,63].

Thermistors are composed of semiconductors materials that change their resistance in response to temperature variations. The relationship resistance-temperature is described by a non-linear law, as expressed by the following equation:

(2) $$R=R_{0}e^{\beta \left(\frac{1}{T}-\frac{1}{T_{0}}\right)}$$

where R_0_ and T_0_ represent the initial resistance and temperature conditions, β is a constant characteristic of the semiconductor employed, and R is the resistance measured at temperature T. These transducers can be classified in two different categories: (i) thermistors with a Positive Temperature Coefficient (PTC), which exhibit a resistance increment because of an increase in temperature; (ii) thermistors characterized by a Negative Temperature Coefficient (NTC) responding by decreasing resistance when a temperature increase occurs [64]. Among these two categories, NTC thermistors are the most popular ones. In the field of HTs, the first application of thermistor dates back in 1976 [65] when researchers developed a thermal probe embedding one thermistor to evaluate the effect of RF fields in biological tissue. Due to their cost-effectiveness, small size, robustness, high sensitivity, fast response time, and good accuracy, further studies have adopted these thermometers to measure temperature in HTs [66,67,68,69,70,71]. It is worth pointing out that both thermocouples and thermistors can provide a temperature measured in a single point of the tissue. Therefore, their use allows gathering temperature information in a specific anatomical point [39].

Fluoroptic and FBGs sensors belong to the large optical sensors’ category. In the last decades, optical sensing technologies have gained momentum in various biomedical applications due to their remarkable metrological properties [72,73,74,75,76,77]. Specifically, the fluoroptic sensor consists of a fluorescent material bonded to a fiber optic tip, representing the fiber’s sensing portion. Their principle of work relies on the decay time of the fluorescent material (e.g., ruby, alexandrite, thulium, rare-earth phosphors) [74,75]. When a light pulse travels inside the fiber, such material is excited. The fluorescent signal resulting from the excitation travels back in the fiber. After the excitation, the signal decays with an exponential low depending on the fluorescent material’s temperature, as reported in the following equation [78]:

(3) $$I_{P}=I_{0}e^{\frac{-t}{\tau }}$$

where I_0_ is the initial intensity value, t is the time, τ the time constant, and I_p_ the emitted signal intensity. From this equation, it is possible to estimate temperature since its dependency on τ. In ablation treatments, the first exploits of fluoroptic sensors appeared in the 90s and viewed as the main application the treatment of cardiac arrhythmias [79,80,81]. After these early investigations, substantial studies reported the use of fluoroptic sensors in liver, prostate, and breast cancer [82,83,84,85]. The popularity of fluoroptic sensors is mainly due to their non-toxicity, biocompatibility, small size, immunity to electromagnetic fields, wide measuring range (generally 25 °C–300 °C), and high accuracy (around 0.2 °C) [74,78]. Nevertheless, it should be kept in mind that fluorescence thermometry may cause measurement errors due to the sensor overheating during the HT [39].

FBGs were introduced for the first time in 1978 [86]. Basically, an FBG is an intrinsic optical sensor inscribed inside the core of an optical fiber consisting of a fiber’s portion in which a periodic modulation of the refractive index occurs. The incidence of a broadband light source on the grating results in a reflection of a narrow spectrum centered around a specific wavelength (i.e., the Bragg wavelength λ_B_) satisfying the Bragg condition expressed in the following equation [87]:

(4) $$\lambda_{B}=2\cdot n_{\mathrm{eff}}\cdot \Lambda$$

As highlighted by Equation (4), λ_B_ value is determined by the effective refractive index (i.e., n_eff_) and the grating period (i.e., Λ). λ_B_ shift (∆λ_B_) occurs when the fiber is exposed to strain or temperature variations. Thus, by measuring ∆λ_B_ it is possible to retrieve the strain, or temperature variations. In HTs, FBGs are used as temperature sensors only by implementing strain free-configurations or adequate filtering stages, especially for vivo trials where breathing-related organ movements can potentially cause measurement artifacts [88,89]. FBGs have found applications in RFA [90,91,92,93], LA [94,95,96,97], MWA [98,99,100,101] and HIFU [102]. The overgrowing number of studies employing FBGs to perform temperature measurements is explained by several advantages making them more powerful than other thermometric techniques. In addition to the features already listed for fluoroptic sensors, FBGs are recognized for their multiplexing capability, allowing multiple sensors within a single fiber [103]. Thus, knowledge about heat distribution in the surrounding tissues can be easily gathered. Anyway, the interrogation system required to both power the fiber and retrieve data makes optical sensors very expensive.

#### 2.1.2. Contactless Techniques

This category involves diagnostic imaging techniques (i.e., MRI, CT and ultrasound thermometry), allowing a temperature mapping reconstruction during the procedure [39]. CT scan is a popular diagnostic imaging method employed in clinical practice to examine body sections for the diagnosis of several diseases. The digital imaging resulting from a CT scan is constructed from pixels representing the linear attenuation coefficients of the examined tissues, which are processed and converted into CT numbers (expressed in Hounsfield Unit, HU) obtaining from the following equation [104]:

(5) $$\mathrm{CT}\left(x,\;y\right)=\frac{1000\cdot \left[\left(\mu \left(x,\;y\right)-{\;\mu }_{W}\;\right)\right]}{\mu_{W}}$$

where μ_W_ is the water linear attenuation coefficient and μ(x,y) is the average linear attenuation coefficient in the (x,y) voxel. The linear attenuation coefficient depends on tissue-X ray interaction, resulting from Compton effect. This phenomenon is mainly affected by tissue density (ρ) which inversely depends on temperature variation as evidenced by the following equation [105]:

(6) $$\rho \left(T\right)=\frac{\rho \left(T_{0}\right)}{1+\alpha \cdot ∆T}$$

where ∆T is the temperature variation, T_0_ is the room temperature and α is the thermal expansion coefficient. Using a Taylor series linearization, it is possible to estimate temperature by CT numbers. CT thermometry appeared in HTs in the early of 1980s [106,107,108]. Since its first application, studies employing this technology are not lacking [55,109,110,111,112,113,114,115,116]. CT thermometry exhibits good spatial resolution, a fast acquisition time if compared to other diagnostic techniques (e.g., MR) and a temperature precision around 3 °C. Whereas, one concern is the radiation dose [117]. 

During ultrasound imaging, a high-frequency sound wave is transmitted within the human body by means of a mechanical transducer. The resulting ultrasound image is made up of the echoes generated by the reflection of ultrasound waves. The depth of the examined tissue can be estimated according to the time elapsed between the wave sending and the detection of the reflected wave by the transducer. The time delay can be measured using the below equation [118]:

(7) $$t\;\left(T_{0}\right)=\frac{2z}{v_{s}\;\left(T_{0}\right)}$$

in which t(T_0_) represents the time delay of the reflected wave measured at the depth z and temperature T_0_. When a temperature variation (∆T) occurs because of the medium’s thermal expansion and changes in the sound speed, a time shift (∆t) can be measured. The relationship between ∆T and ∆t can be expressed by the Equation (7), considering negligible the contribution due to the thermal expansion compared to sound speed [119].

(8) $$∆T={\;k}_{1}\frac{d\left(∆t\right)}{\mathrm{dt}}$$

In Equation (8), k_1_ is a tissue constant and the term $\frac{d\left(∆t\right)}{\mathrm{dt}}$ the normalized time shift. Thus, temperature distribution inside the tissue can be obtained by considering the dependence of T on the sound speed. Ultrasound-based thermometry emerged in 1979 [120] and then found broad acceptance in all HTs [121,122,123,124] except for HIFU ablation, where the employment of this method should be avoided to prevent cross-talking phenomena [125]. In addition to its non-invasiveness, this technique does not require ionizing radiation and is quite inexpensive as compared to other imaging techniques. However, this thermometry may be affected by measurement artifact due to the patient’s breathing and motions [39].

MR provides diagnostic images by applying an external magnetic field. Hydrogen protons are mainly responsible for the diagnostic image resulting from an MR scan. These protons and their electric charge revolve around an axis (usually said to have spin). When a high external magnetic is applied (B_0_), the proton axis will be oriented along the field itself. The orientation can occur in the same direction of B_0_ (i.e., configuration with low energy) or in the opposite one (i.e., configuration with high energy level). In addition, in presence of B_0_, the axis of each proton revolves with respect to B_0_, at a defined frequency, called precession frequency. To resonate the hydrogen proton, a radiofrequency signal with frequency equal to the precession one is applied. After its interruption, the proton spins return to their initial condition. This phenomenon can be described by two time-related parameters (i.e., spin-lattice relaxation time, T1 and longitudinal relaxation time, T2) [126]. Several MR parameters exhibiting temperature dependence have been investigated [127]. Among others, T1 and proton resonance frequency (hereafter PRF) were found to be most promising [128]. The relationship between T1 and temperature is expressed by the following equation [129]:

(9) $$T1\propto \exp\left(-\frac{E_{a}\left(T1\right)}{k\cdot T}\right)$$

where $E_{a}\left(T1\right)$ denotes the activation energy of the relaxation process, k the Boltzmann constant and T is the absolute temperature. The relationship described can be considered almost linear for temperature ranging between 30 °C and 70 °C [39]. The main drawback related to the use of this technique is the T1 dependence on the tissue that may affect the measurement. Another parameter used to measure temperature is the PRF. This technique overcomes limitations due to the tissue type dependence and shows high precision in measuring temperature variations. In this case, ∆T is related to the image phase according to the below equation [130]:

(10) $$∆T=\frac{\varphi \;\left(T\right)-\varphi \left(T0\right)}{\gamma \cdot \alpha_{1}\cdot B_{0}\cdot \mathrm{TE}}$$

where α_1_ is the PRF variation coefficient, TE is the echo time, γ is the hydrogen gyromagnetic ratio, φ (T) and φ (T_0_) the image phases at temperature T and T_0_, respectively. In 1988, MR thermometry found application for the first time in LA [131]. Subsequently, substantial research about MR-guided temperature monitoring during HTs was carried out [83,132,133,134,135,136,137,138,139].

### 2.2. Temperature Monitoring during RFA in Bone

After a short description of the RFA principle of work, we explore the main studies focused on temperature monitoring during RFA. 

RFA differs according to the working modality (i.e., monopolar, or bipolar). In monopolar mode, a high-frequency alternating current (typically between 350 kHz and 500 kHz) [38] is delivered into the tumor tissue by means of an electrode with an exposed tip (i.e., the active electrode). A RF generator produces a voltage difference between the active electrode and a ground pad placed on the patient’s skin. The applied current causes ionic agitation in the tissue around the exposed tip; as a result, tissue heating due to the Joule effect occurs [24]. In bipolar mode, the current flow is established between two electrodes both in contact with the tissue, and no ground pad is required [140].

The first application of bone RFA in a clinical trial dates back to 1992, as reported in a scientific article published by Rosenthal et al. [141]. This study reported four case studies of patients affected by OO in different anatomical sites and treated with RFA. After these early investigations, RFA has won extensive clinical acceptance in the treatment of bone tumors [26,142,143,144,145,146,147].

Among a huge number of studies focused on bone RFA, many of them have also investigated temperature monitoring. 

In [43], Dupuy et al. carried out RFA on both in vivo pigs and ex vivo samples by performing simultaneous temperature assessment during treatment. Five pigs underwent RFA after being anesthetized. The procedure was performed in lumbar vertebral bodies (L1-L3) and paraspinal muscles (total of 10 procedures, 5 for each anatomical site). Three holes were drilled into the vertebral body using a biopsy needle to position RF probe and three thermistors. The treatment was carried out for an application time of 12 min at the maximum current and 1 cm of the active tip. The three thermistors were positioned adjacent to the spinal canal at 5 mm, 10 mm, and 15 mm from the probe. Temperature values were recorded each 5 min during and after the RFA until the temperature return to the initial value. During ex vivo experiments, samples of cancellous and cortical bone were subjected to RFA for 6 min using an RF probe with 2 cm of the active tip. In these experiments two thermistors were positioned at 10 mm away from the tip, in both bone sides in case of cortical bone. Results showed differences in temperature values recorded inside the vertebral body compared to those in the paraspinal muscle in correspondence of 10 min (i.e., lower values in the first case than in the second at the same distances). In the epidural space temperature was equal to 44 °C. Temperatures assessed in ex vivo evidenced an insulating effect of cortical bone. Indeed, at the same distance of 10 mm but on opposite sides of bone samples, thermistor in the proximity of bone cortex registered higher value than the one positioned in the cortical bone (i.e., 25.7 ± 7.0 °C and 11.2 ± 2.0 °C, respectively).

In [148], the RFA feasibility in bone was assessed by estimating the heat distribution both in cortical bone and marrow in ten ex vivo bovine tibia. Five holes of 2 mm in diameter were drilled in the tissue to allow thermocouples insertion parallel to the RF electrode. The holes were cut at several depths and a distance of 5 mm radially. Twelve measurements were performed for each distance, and temperature values were recorded every 1 s. RFA was served with a cool-tip probe for a maximum application time of 30 min. In the bone marrow, mean temperature values of more than 50 °C were found at distances of 5 mm, 10 mm, and 15 mm. In correspondence of 20 mm, an exposure time of 30 min was not sufficient to reach 50 °C. In cortical bone, temperatures were lower than those experienced by bone marrow at the same positions (i.e., probe tip, 5 mm and 10 mm).

Temperature changes in tissue surrounding bone during RFA of OO were also investigated in [149]. Experiments were conducted in ex vivo animal model (i.e., excised bovine tibia). The OO nidus (i.e., the core of tumor mass) was mimicked by a hole filled with agarose. Based on the thickness of cortical bone between the periosteum and the nidus (i.e., 1 mm, 3 mm, and 5 mm), the authors distinguished three groups, each accounted for three specimens. Three thermocouples were inserted into each sample at the following positions: (*i*) in direct contact with the periosteum, (*ii*) at 5 mm, and (*iii*) 10 mm from the periosteum. The RFA was performed by setting a target temperature to 95 °C and a treatment time of 400 s, and temperatures were recorded at regular intervals of 10 s during the procedure. Results revealed an influence of cortical bone thickness on heat propagation. Indeed, the highest temperature values (up to 69.3 °C) were recorded in the case of 1 mm-cortical thickness in all three measurement sites when compared with the other two groups (59.2 °C for 3 mm thickness and 50.6 °C for 5 mm thickness). This study highlights how a specific anatomical parameter may impact on temperature distribution, thus on the irreversible thermal injury in the surrounding structures.

In [150], the safety of RFA was evaluated by measuring the temperature inside lumbar vertebral bodies both in the presence and in the absence of a cortical bone defect. The evaluation was performed on both in vivo and ex vivo experiments. In the first case, ten lumbar vertebral bodies of six porcine adults were subjected to RFA with an active tip of either 1 cm or 2 cm. Three k-type thermocouples were used to monitor the temperature every 30 s in the intervertebral foramen, within the vertebral body, and at the frontal vertebral surface. Differently, two thermocouples were employed in ex vivo experiments. In this case, the authors evaluated heat distribution in six lumbar vertebral bodies excised with and without cortical bone. Thermocouples were allocated so temperature could be measured within the spinal canal and in the paravertebral area at regular intervals of 30 s. The in vivo study allowed comparing temperatures in the spinal canal in case of 1 cm active tip electrode with those recorded during RFA carried out with a 2 cm active tip probe. Differently, ex vivo experiments were carried out to compare temperature values during RFA in tissue samples with and without cortical defect. Specifically, during in vivo tests, the temperature recorded was much higher in the RF probe with 2 cm of the active tip, especially for the spinal canal and vertebral body. Ex vivo experiments pointed to higher temperatures in cortical bone defect samples, most notably in the spinal canal. The authors proved the risk for irreversible thermal damage occurring in nerve structures in both case studies.

In [151], authors aimed at evaluating the usefulness of real-time temperature monitoring within the spinal canal for bone tumors contiguous to the marrow. The study included ten patients with spinal metastases (i.e., 3 in thoracic spine, 6 in the lumbar, and 1 in the sacral area) resulting from previous primary tumors. All tumors were in proximity of the spinal cord (mean distance of 2.4 ± 1.6 mm). A thermocouple positioned between the tumor mass and the spinal cord (precisely in the epidural space in six cases and in the subarachnoid space in four others) allowed monitoring temperature in real-time at its tip since it was connected to an external monitor. In all procedures but one, the temperature did not exceed 45 °C. In a single case, thermocouple recorded 48 °C causing a temporary neural injury for the patient fixed by steroid injection. The study evidenced the importance of temperature monitoring as a key method to prevent acute complications in lesions involving the spinal cord.

In [152], temperature was monitored inside the human cadaver vertebrae by three k-type thermocouples. The aim was to compare three different ablation devices (two different RF probes, 20 mm array, and 10 mm single electrode, and one coablation device). The thermocouples were positioned under fluoroscopic guidance in the vertebral body, epidural space, and neural foramen. Temperatures recorded at the end of the treatment revealed the safety of coablation to treat metastatic lesions located in vertebral body.

In [153], temperature was measured by a thermocouple positioned parallel to a bipolar RF probe in an ex vivo vertebral body. The thermocouple allowed verifying that temperature outside the target tissue was keeping around the physiological value.

In [154], RFA was carried out on long bovine bone cut into 5 cm and 10 cm pieces within which holes were drilled into the cortical and cancellous bone to simulate OO’s cavity. The created cavity was filled with homogenized liver and agar to mimic the nidus of the tumor mass. Temperatures were measured at a sampling frequency of 1 Hz using thermocouple embedded within a multiple RF probe at different distances (from 2 mm to 14 mm) from the tumor edge. For tumors less than 10 mm in diameter, temperatures recorded in cancellous bone were higher than those in cortical bone for the same size tumor and distance. Through a multiple regression analysis, a predictive model to estimate temperatures in cortical and cancellous bone was defined as follow:

(11) $$\begin{array}{c} T=43.051+1.965\times D-17.335\times \log\;\left(\mathrm{DFE}\right)\\ T=72.249+2.66\times D-47.246\times \log\;\left(\mathrm{DFE}\right) \end{array}$$

where T is predicted temperature in cortical (for the first equation) and cancellous bone (for the second equation), D is the tumor diameter in mm, and DFE is the distance between the tumor edge and the temperature measurement site. This study paves the way for a clinical tool which may enable treatment planning for patients with OO.

In [45], temperature distribution during RFA in ex vivo vertebral bodies was evaluated to test a new spinal ablation device. Sixteen vertebrae (i.e., 8 lumbar and 8 thoracic vertebrae) collected from human cadavers were subjected to RFA. In this study, temperature measurements were accomplished by means of three k-type thermocouples placed inside the vertebral body, in the neuroforamen, and the spinal canal and recorded every 30 s. The results showed no significant differences in terms of temperature recorded in lumbar versus thoracic vertebrae. A similar study was reported by the same research group [155], aiming at comparing three different kinds of RF probe.

In [156], ex vivo and in vivo experiments allowed evaluating the effect of heat distribution in the spinal canal and in the surrounding areas of vertebral bodies during RFA. RF probe consisted of a needle equipped with 13 hooked electrodes (see Table 1). The first studies were performed in vitro by means of thirty ex vivo swine vertebral bodies divided in two groups: (i) a first group in which RFA was accomplished inserting the electrode tip at a depth of 10 mm and (ii) a second group where the tip was introduced for 20 mm. Before and after the procedure, temperature changes were recorded at 10 mm and 20 mm from the RF probe and in the front wall of the spinal canal and the ventral side of the vertebra. Differently, for in vivo assessment, two pigs were subjected to RFA in the lumbar vertebral body. Under imaging guidance, thermistors were placed in three different vertebral body sites: (i) at a depth of 10 mm; (ii) posterior area; and (iii) lateral area. Temperature values were recorded every 5 min and up to 20 min during the procedure. Results obtained for in vitro trial revealed temperatures higher in Group 2 than Group 1, with a value up to 50.8 °C in the spinal canal at the end of the ablation. Moreover, temperature recorded at 20 mm from the probe was higher than the ones showed for 10 mm (i.e., 37.7 ± 2.0 °C and 33.7 ± 1.7 °C, respectively). In vivo experiments showed temperature values always lower than 45 °C (i.e., cytotoxic threshold) in the posterior and the lateral areas, thus avoiding spinal cord impairment.

In [51], a thermocouple was inserted into the epidural space to monitor temperature variations in the anatomic area lying between the posterior site of the vertebral body and the dura mater. RFAs were performed in thirteen patients affected by lumbar tumors. During the treatment, temperature monitoring was carried out in combination with hydrodissection (in 11 cases). The temperature provided by the thermocouple was used to control in real time the RFA procedure by turning off the power when temperature reached 45 °C. The knowledge of temperature evolution in real-time allowed the preservation of the vulnerable structures close to the tumor mass. Also, the same research group reported a study involving 31 patients suffering from spinal metastases treated with RFA [157]. In this case, temperature monitoring was performed by means of a thermocouple positioned at the level of the vertebra’s posterior wall or into the epidural space. This study aimed at evaluating the efficacy of bipolar radiofrequency ablation with a target temperature of 70 °C. A study similar to [51] was carried out by the same research group in 2021 [158]. In this article, the authors suggested a combined technique of temperature monitoring and hydrodissection within the anterior epidural space in seven patients affected by thoracic metastases. To record temperature, authors used a thermocouple integrated into the RF probe. In all the procedures performed, temperature did not exceed 45 °C. 

Table 2 summarizes studies performing temperature monitoring during RFA in bone.

### 2.3. Temperature Monitoring during LA in Bone

In this subsection, the LA working principle and its first application in bone tumors will be briefly reported. After this introduction, studies referring to temperature monitoring during LA will be described in depth.

Basically, LA involves the use of a laser source and an optical fiber (around 200 μm in diameter) which is responsible for carrying the light within the tissue. A monochromatic light is emitted at a specific wavelength by a laser. The wavelength employed attributes the laser properties and the manner of interaction with the tissue. Light-tissue interaction takes place through three different phenomena: scattering, reflection, and absorption [159]. The absorbed light is mainly converted into the heat. The amount of heat produced within the tissue is affected by several factors. Among others, there are the laser wavelength, laser working modality (i.e., continuous or pulsed), treatment time, input power, and physical and optical tissue properties [160]. Commonly employed systems consist of 980 nm diode laser and 1064 nm neodymium-doped yttrium aluminum garnet (Nd: YAG) which guarantee optimal absorption and penetration rate.

LA was firstly introduced for OO treatment in 1998 by Gangi et al. [161]. Later investigations carried out during a clinical trial can be found in the literature [11,162,163,164].

Only few studies investigated temperature monitoring during LA. In 2002, Binkert et al. [165] evaluated the possibility to monitor temperature in real-time with MR thermometry. Two pigs were subjected to LA, for a total of nine vertebrae treated. Nd: YAG system at an input power of 10 W and a treatment time of 2 min was employed to accomplish the procedure. MR-thermometry was carried out using the T1 method because of its good sensitivity in the presence of low electromagnetic field (in this study 0.5 T) and its ability to track temperature in the spinal cord. In MR images, thermal necrosis was found since 20 s after applying energy and it could be observed by an increase in color brightness. Irreversible T1 signal changes were observed in the middle of the treated area, in the paraspinal muscle and in the spinal canal because of attaining cytotoxic temperatures. Thermal injuries in the spinal cord occurred in presence of cortical bone defects. In this study, authors demonstrated the potential of MR-thermometry to keep track of thermal injuries in the vertebral bodies and spinal canal. It is worth noting that the acquisition of thermal map was obtained only during apnea to avoid motion artifacts due to respiratory movements.

In [166], MRI scanner in a low-field configuration (0.23 T) was employed in five patients undergoing LA (Nd:YAG). Unlike previous studies, authors revealed an unreliable change in T1 relaxation time in response to temperature increment because of weak signal from cortical bone, accounting for the majority of the OO.

Few years later, Streitparth et al. [167] published results of a clinical trial involving one patient affected by OO. LA was carried out by Nd:YAG laser at 2.3 W of power and an operating time of 11 min. Additionally, MRI scanner (1.0 T) was used as both guidance and to monitor T1 changes due to temperature increment. A commercially available software allowed displaying in real-time temperature map and tissue alterations. No further details regarding temperature values reached in the tumor or in the surrounding healthy structures were given in this investigation. Also, the same research group employed MR-thermometry based on T1 method with the aim to protect surrounding anatomical structures (i.e., joint cartilage) in patient affected by a recurrence of tibial OO [168].

A retrospective study conducted by Tatsui et al. [169], evaluated the feasibility of treating spinal tumors with LA. For this purpose, 11 tumors located in thoracic, lumbar, and cervical spine with epidural space involvement were treated with a 980-nm diode laser (at an input power of 30 W). Temperature monitoring was accomplished by PRF method. An ad-hoc software allowed retrieving temperature values in each image’s pixel. A control-algorithm enabled to stop energy delivery when the temperature between the epidural space and the dura mater exceeded 50 °C. This method prevented irreversible damage in structures such as spinal cord and nerve roots. Whereas breathing-related movements can affect temperature estimation, laser was switched on during apnea phase, thus avoiding possible artifacts in thermal map resulting from MR-thermometry. One year later, the method just described was used by the same research group on a larger cohort of patients (i.e., 19) affecting by spinal metastasis with epidural compression [170]. 

A brief summary of the studies performing temperature monitoring in bone during LA is provided in Table 3.

### 2.4. Temperature Monitoring during MWA in Bone 

After a short introduction about MWA, this subsection will be devoted to describing studies performing temperature monitoring during MWA in bone.

During MWA, electromagnetic radiations (usually with a frequency of 915 MHz or 2.45 GHz) are conveyed within the tissue by means of the so-called antenna. Dielectric heating occurs due to the interaction between water molecules and the applied field. So, in presence of an alternating field, dipoles revolve continuously to align with it. This phenomenon results in the production of frictional energy leading to temperature increment [171]. Compared to other HTs, MWA allows temperatures above 100 °C, increased ablation volume and shorter treatment time.

The first scientific investigations on MWA in bone go back to 1996 [172,173]. Due to the encouraging results obtained in these works, MWA received broad clinical acceptance for the treatment of bone tumors [28,29,174,175,176,177].

Studies concerning temperature monitoring during MWA in bone are lacking in the literature, and the few research articles found are quite recent.

In 2014, temperature monitoring was performed during MWA of spinal metastasis [178]. The study included seventeen patients, treating a total of twenty lesions. MW ablation system was set to deliver a power between 30 W and 70 W, applied for a variable duration ranging from 1 min to 8 min, depending on the lesion size. Only in four cases, a thermocouple was used to monitor in real-time temperature variations because of the proximity to neural structures. No additional details were given about temperature values recorded during the procedure.

A mention of temperature monitoring is also provided in [179]. In their study, authors cited the use of multiple thermocouples placed at critical anatomical sites to monitor temperature in and around the target area, during MWA in malignant bone tumors.

In [180], a thermocouple was used for temperature monitoring during MWA of bone metastases aiming at preventing irreversible thermal injury to nearby structures. At this purpose, a clinical trial involving 16 patients suffered from secondary bone tumors was carried out, for a total of 18 MWAs. The procedures were performed with an input power of either 15 W or 40 W and a treatment time ranging between 1 min and 6 min. According to the cancer mass positioning (spinal column, rib and sternum), the thermocouple was placed in the following anatomical sites: epidural space, nerve roots, pleura (in case of rib ablation) and pericardium (during sternal ablation). In three procedures, more than one thermocouple was inserted in the treated area to control temperature in different neural structure’ sites. In eight cases MWA was interrupted due to the occurrence of cytotoxic temperatures. 

For the first time, FBGs appeared for bone temperature monitoring purpose in [181]. MWA was carried out in ex vivo bovine bones (i.e., femur and tibia) setting a power of 75 W and ablation time of 8 min. Temperature monitoring was accomplished by means of four fiber optics, each embedding 10 FBGs. Their use allowed measuring temperature in 40 different anatomical sites. Maximum temperature values (i.e., 63.2 °C in case of femur ablation and 91.4 °C during tibia MW ablation) were recorded by one of the FBGs belonging to the optical fiber positioned closer to the MW antenna at the end of the treatment in both ablation procedures. In a post-processing phase, the multi-point temperature measurements allowed obtaining temperature map by a linear interpolation of the temperature data recorded during femur and tibia ablation. The resulting thermal maps helped to gain useful information regarding the heat distribution not only near the treated area but also in the surrounding ones. This work was the result of a deeply investigation after a preliminary assessment carried out by the same research group in shortly before [182].

Recently, a retrospective study carried out by Ke et al. [183] presented results obtained for 56 bone metastases underwent image guided MWA. One or more thermocouples were employed to monitor temperature in different anatomical sites. The use of these thermometers was intended as a control for ensuring temperature values below 43 °C in healthy tissues.

A short summary of the applications related to temperature monitoring during MWA in bone is provided in Table 4.

### 2.5. Temperature Monitoring during HIFU in Bone 

In this subsection we will give a quick description of the HIFU’s working principle and then we will describe studies performing temperature monitoring during HIFU ablation in bone. 

Differently from the previously described techniques, HIFU ablation does not require surgical incision or needlelike probe insertion for the treatment of tumor masses. It involves the use of high-focused ultrasound (frequencies ranging between 0.2 MHz and 3.5 MHz) converging in a specific point (i.e., focal point) where the energy is highly concentrated by means of a transducer. The energy conveyed is able to promote irreversible biological tissue damage within the focal point, because of temperature raising at cytotoxic levels [184]. A feasibility assessment about the efficacy of HIFU for the treatment of primary or secondary bone tumors appears in 2001 [185]. Despite its late application in bone tumors, multiple studies showed promising results concerning the application of HIFU in this hard tissue [33,35,186,187,188,189,190]. 

Other relevant studies have also explored temperature monitoring using MR-thermometry. A mention of thermal maps acquired during MR-guided HIFU ablation of OO can be found in [191]. 

In [192], an investigation was carried out to evaluate the temperature dependence of cortical bone tissue during MR-guided HIFU ablation. In a first phase, experiments were carried out in ex vivo beef shanks to preliminary assess temperature changes in cortical bone under MRI. At this purpose, bone tissue was heated in a hot water bath and after subjected to MR-scanner (3.0 T and a short TE gradient echo imaging sequence) to record temperature changes during the cooling phase. Fiber optic sensors previously inserted in cortical bone was used as reference system. Phase image changes were fitted with temperature recorded from fiber optic sensors, showing a good correlation between the two set of data (R^2^ > 0.85). A subsequently evaluation consisted of MR-HIFU (1.2 MHz transducer) of an ex vivo beef shank immersed in a gum phantom. Once again, four fiber optics placed inside the cortical bone were used to assess the reliability of temperature data estimated by PRF method during MRI. In a post-processing phase, thermal map of bone tissue was also derived by means of a dual echo approach (i.e., short and long echo acquisition) to obtain temperature information about both bone and soft tissue.

Lam et al. [193] evaluated the performance of PRF shift method during a clinical trial enrolling eleven patients subjected to HIFU treatment in bone (i.e., osteolytic, osteoblastic and mixed lesions). This study aimed at assessing the image quality in terms of signal-to-noise-ratio (hereafter SNR) and temperature variations resulting from phase image changes and to score artifacts derived from: field inhomogeneities, arterial ghosting and patient motion. Each treatment was carried out with variable treatment settings (i.e., power ranging from 20 W and 50 W and time between 16 s and 20 s) and 1.5 T-MR scanner. Results revealed a highest SNR in case of osteolytic lesions and a maximum temperature variation up to 1.8 °C in mixed lesions. About artifacts, field inhomogeneities related to breathing was found to be the most dominant, thus resulting in a potential temperature offset.

In [194] T2-based thermometry was used to measure temperature variation in the spinal cord of ex vivo and in vivo bovine bone during MR-HIFU ablation. After a temperature calibration process, two ex vivo bovine femur were cut to expose the trabecular tissue to the ultrasound beam. HIFU system worked at a frequency of 500 kHz and an acoustic power of 17.6 W for a treatment duration of 8 min. Three fiber optic sensors were inserted inside the spinal cord as reference system. T2 values were measured in three different ROI (i.e., in correspondence of the fiber optics tips). Another ex vivo experiment consisted of HIFU ablation (1.15 MHz-transducer, acoustic power of 30 W and sonication time of 20 s) in an intact ex vivo swine femur to mimic the real condition. HIFU ablation was also performed in in vivo swine model. For each of these experiments, thermal tissue map was retrieved. Despite in all cases the focal point was conveyed within the bone marrow, in ex vivo trabecular ablation, the high temperature values were recorded in bone tissue because of its high ultrasound absorption rate. Also, a linear relationship between temperature values recorded by the fiber optic and the T2 changes was found in the heating process. For intact ex vivo ablation, an increment of T2 values was obtained in the marrow. Finally, for in vivo test the maximum T2 value was recorded at the end of the treatment and a value of 231 ms was found in the bone marrow corresponding to a temperature variation of 33 °C.

In [195], nine patients affected by OO were treated with MR-HIFU ablation. The ablation system consisted of an ultrasound transducer and 1.5 T MR-scanner. Treatment settings were changeable according to the lesion size. MR-thermometry in the affected tissue portion and in the closest tissues were carried out to ensure the safety of the procedure. Thermal maps resulting from the procedures evidenced similar temperature values in the surrounding anatomical structures and no tissue heating occurred beyond pre-planned treatment margins.

Study published by Guillemin et al. [196] consisted of a MR-guided HIFU procedure in an ex vivo animal tibia drilled to mimic osteolytic tumor mass. Experiments were performed with an acoustic power of 60 W and temperature monitoring in tissue adjacent to the periosteum was accomplished with the PRF shift method employing a 3T MR-scanner. Due to the lack of MR-signal in cortical bone, temperature in this anatomical structure was monitored by means of a fluoroptic sensor positioned inside it after drilling. Experiments were carried out resembling different focal point positioning (i.e., inside the medullar cavity, in front of the medullar cavity and in the cortical breakthrough). Procedure’s safety was evaluated by matching data retrieved from MR-thermometry and fluoroptic sensors. A predictive temperature model was developed to tune the acoustical energy deposition automatically with the aim of controlling temperature rise at the focal point. Finally, a numerical simulation allows estimating the time delay between the energy application and the temperature rise at the focal point.

In [197], authors demonstrated the possibility to monitor temperature in water and fat in the treatment of bone lesions during MR-guided HIFU. MR-thermometry was conducted using alternately the PRF and T1 methods. The proposed solution was evaluated both in ex vivo swine leg and in a healthy volunteer without heating conditions. Thermal maps gained allow obtaining temperature information in water and fat voxels by overlapping information obtained from PRF and T1 thermometry. Thus, providing information in districts surrounding the bone and not limited to the treated portion.

Table 5 summarizes studies performing temperature monitoring during HIFU in bone.

## 3. Discussions and Conclusions

In the last decades, HTs are gaining momentum in bone malignancy management because of both encouraging results in terms of pain relief affecting a wide cohort of patients and indisputable advantages of these minimally invasive techniques. It is worth noting that among innovative areas of research involving HTs, its use in synergy with conventional treatments (e.g., chemotherapy or radiotherapy) may overcome one of the most important concerns of HTs which is the incomplete tumor destruction [198]. In this scenario, temperature monitoring may be beneficial to enhance abscopal effects which have shown promising results in advanced cancers [199,200,201]. 

During bone ablations and more generally in such kind of treatments, one of the major pitfalls concerns either the prediction or the real time knowledge of the effects. Thermal injury occurs because of the existence of cytotoxic temperatures inside the tissue and exposure time. Thus, temperature tissue monitoring may be helpful to ensure the complete tumor destruction while sparing healthy anatomical structures. This aspect deserves special attention in bone malignancies owing to the presence of vulnerable structures (i.e., spinal cord and nerve roots). Indeed, temperatures above 45 °C would be neurotoxic for such structures and could potentially lead to permanent impairment. In the light of above, in this work, we focused on the potential of temperature monitoring during bone HTs. Studies investigating temperature measurements in bone were reviewed and reported according to the specific HT employed (i.e., RFA, LA, MWA and HIFU). Pre-clinical and clinical studies were found to explore the applicability of specific thermometric techniques tailored to this specific scenario. Among contact-based and contactless techniques used to record temperature during HTs, only some of them were adopted in this specific context. Thermocouples, thermistors, and MR-thermometry play a leading role during HTs in bone. Very few studies addressed the potential of FBGs for temperature measurements purposes during bone ablation, despite their popularity in other hyperthermia applications. Otherwise, fluoroptic sensors were only used in the validation of MR-thermometry during bone HIFU procedures. To the best of our knowledge, to date, literature lacks investigations regarding CT and ultrasound thermometry in bone ablation. From non-exhaustive inferences, contactless techniques could be expected preferably in bone ablation context where preserving vulnerable structures is a priority. Unfortunately, despite this category of techniques is capable of reconstructing temperature tissue map, it is not immune to drawbacks which severely limits its use. Of course, the use of sophisticated algorithms to estimate temperature and the high costs of diagnostic imaging techniques are two of the negative issues to noteworthy. In case of CT-thermometry the radiation dose is another aspect to be kept in mind. Furthermore, contactless thermometry is affected by measurement artefacts due to patients’ movements, especially those due to breathing, hence the need to implement alternative solutions to overcome this concern (e.g., signal acquisition during breathing holding, algorithms devoted to artifact removal). In the specific case of MR-thermometry in bone, it should be pointed out the lack of MR-signal in cortical bone which may lead to unreliable and inaccurate temperature measurements. Unexpected, contact-based techniques are so far well suited to the context. The broad implementation of transducers such as thermocouples and thermistors are mainly due to their low cost, small size, robustness, wide measuring range, short response time and ease to use which make them preferable to other techniques involving a high level of expertise. Despite their invasiveness, many studies exploring temperature monitoring in bone employed such kind of solution offering the right balance between affordability and reliability. Also, the use of these techniques overcomes the issue of breathing-related artifacts. On the other hand, thermocouples and thermistors provide a single-point measurement, thus it is not feasible to obtain temperature map for estimating thermal tissue damage. Moreover, owing to their metallic composition, these thermometers cannot work in presence of high electromagnetic fields (e.g., MR). Although FBGs are currently lacking special attention in the field of bone ablation, they appear very promising in this arena because of their countless features, among others biocompatibility, small size, immunity to electromagnetic fields, wide measuring range and high sensitivity. A special mention deserves their multiplexing capability which allows temperature measurements in several point with high resolution (even less than 1 mm) and an accuracy around 0.1 °C (but strongly dependent to the quality of the interrogator system). Thus, it is possible to obtain reliable temperature map which is the key aspect especially in this specific context where incorrect estimation could lead to irreversible injuries in healthy susceptible areas.

Summing up, from an overview of bone temperature monitoring, the studies found corroborated the importance of this key aspect during bone HTs. Most of these studies aimed at assessing tissue thermal response (e.g., in cortical bone, epidural space, bone marrow) and preventing permanent damage in vulnerable structures, which represents the most challenging aspect of this scenario. Other works investigated the suitability of specific thermometric techniques in monitoring and predicting temperature under particular settings. Only some explored the performances of specific devices in terms of enhancement in safety and clinical outcomes improvement. However, despite many investigations were performed during clinical trials, nowadays, temperature monitoring during bone ablation is still severely restricted in clinical settings. In our view, substantial research efforts are still necessary for making practical some technologies in medical scenarios where clinicians may benefit of being led by real-time temperature knowledge during the procedures without being forced to alter their clinical practice.

## Figures and Tables

**Table 1:** Main benefits and drawbacks of thermometric techniques employed during HTs.

Thermometric Techniques	Benefits	Drawbacks
Thermocouples	Low cost; small size; robustness; wide measurement range; and short response time	Invasive; single point measurement; metallic composition; potential measurement artifacts
Thermistors	Low cost; small size; robustness; high sensitivity; short response time; good accuracy	Invasive; single point measurement; potential measurement artifacts
Fluoroptic sensors	Biocompatibility; small size, immunity to electromagnetic fields; wide measuring range; high accuracy	Invasive; single point measurement; fragility; potential measurement artifacts
FBGs	Biocompatibility; small size; immunity to electromagnetic fields; high accuracy; short response time; multi-point temperature measurements;	Invasive; fragility; cross-sensitivity to strain; high-cost
CT-thermometry	Non-invasive; thermal map reconstruction; good spatial resolution; fast acquisition time; temperature precision around 3 °C	Ionizing radiation dose; potential measurement artifacts; quite expensive
US-thermometry	Non-invasive; thermal map reconstruction; absence of ionizing radiation; quite inexpensive	Potential measurement artifacts
MR-thermometry	Non-invasive; thermal map reconstruction; absence of ionizing radiation; linear relationship between T1 and temperature variations in the range of 30 °C and 70 °C; no tissue type dependence for PRF method	Potential measurement artifacts; lack of MR signal in cortical bone; expensive

**Table 2:** Studies performing temperature monitoring in bone during RFA.

Authors, Reference and Year	Type of Study	Type of Sensors (Number of Sensors)	Type of Probe
Dupuy et al. [43], 2000	Ex vivo and in vivo animal trial	Thermistors (3)	Monopolar RFA
Rachbauer et al. [148], 2003	Ex vivo trial	Thermocouples (5)	Water-cooled single RF electrode (Radionics Instruments Inc.)
Bitsch et al. [149], 2006	Ex vivo trial	Thermocouples (3)	Monopolar RF electrode (TCM 101; Stryker Leibinger, Freiburg, Germany)
Adachi et al. [150], 2008	Ex vivo and in vivo trials	K-type thermocouples (3 during in vivo and 2 during ex vivo experiments)	17G monopolar cooled RF electrode
Nakatuska et al. [151], 2009	Clinical trial	Thermocouple (1)	17G monopolar cooled RF electrode
Groetz et al. [152], 2013	Ex vivo human trial	K-type thermocouples (3)	RFA array electrode (LeV-eenTM Electrode System, Boston Scientific, Natick, USA) Single-needle RFA electrode (Soloist^TM^ Electrode System, Boston Scientific, Natick, USA)
Pezeshki et al. [153], 2014	Ex vivo animal trial	Thermocouple (1)	17G bipolar cooled RF probe (OsteoCool Baylis Medical Company)
Greenberg at al. [154], 2014	Ex vivo animal trial	Thermocouple (not defined)	Monopolar RF probe (ACT-1510 Cool-tip Ablation System, Valley-lab, Boulder, Colorado)
Bornemann et al. [45], 2016	Ex vivo animal trial	K-type thermocouples (3)	Monopolar RF probe (SpineSTAR, DFINE Inc. San Jose, CA, USA)
Bornemann et al. [155], 2016	In vitro model	K-type thermocouples (3)	Bipolar RF ablation electrode (SpineSTAR, DFINE Inc. San Jose, CA, USA) Two monopolar RF electrodes (Soloist and LeVeen, Boston Scientific, Natick, MA, USA)
Wei et al. [156], 2018	Ex vivo and in vivo animal trials	Not specified in ex vivo trial (2) and thermistors in in vivo (3)	Multipolar RFA (RFA-1315, Beijing Bolai, Beijing, China)
Lecigne et al. [51], 2019	Clinical trial	Thermocouple (1)	Bipolar RFA (OsteoCool Medtronic/STAR Merrit Medical) Monopolar RFA (OsteoCool Medtronic)
Mayer et al. [157], 2021	Clinical trial	Thermocouple (1)	Bipolar RFA (Osteocool medtronic)
Lecigne et al. [158], 2021	Clinical trial	Thermocouple (1)	Monopolar RFA (Multigen Stryker, USA) Bipolar RFA (OsteoCool Medtronic/STAR Merrit Medical)

**Table 3:** Scientific articles describing temperature monitoring during LA in bone.

Authors, Reference and Year	Type of Study	Type of Technique	Type of Laser
Binkert et al. [165], 2002	In vivo animal trial	MR-thermometry	1064 nm Nd:YAG
Sequeiros et al. [166], 2003	Clinical trial	MR-thermometry	1064 nm Nd:YAG
Streitparth et al. [167], 2009	Clinical trial	MR-thermometry	1064 nm Nd:YAG
Streitparth et al. [168], 2010	Clinical trial	MR-thermometry	1064 nm Nd:YAG
Tatsui et al. [169], 2015	Clinical trial	MR-thermometry	980 nm diode
Tatsui et al. [170], 2016	Clinical trial	MR-thermometry	980 nm diode

**Table 4:** Studies related to temperature monitoring during bone MWA.

Authors, Reference and Year	Type of Study	Type of Sensors (and Number)	Type of Source
Kastler et al. [178], 2014	Clinical trial	Thermocouple (1)	2.45 GHz-MW generator (Microsulis/AngioDynamics, Latham, New York) and 14 cm or 19 cm long of MW antenna.
Fan et al. [179], 2016	Clinical trial	Thermocouples (not specified)	2.45 GHz MW generator and co-axial antenna (no further details provided).
Kastler et al. [180], 2017	Clinical trial	Thermocouples (1 or more than one in 3 cases)	2.45 GHz-MW generator (AngioDynamics, Inc, Latham, New York) or Amica (Hospital Service, Rome, Italy). Details about the antenna used were not specified.
De Vita et al. [181], 2020	Ex vivo animal trial	FBGs (40)	2.45 GHz-MW generator and 15 cm long antenna with an active tip of 31 mm (Microwave Ablation System, Surgnova Healthcare Technologies, Zhejiang)
De Tommasi et al. [182], 2020	Ex vivo animal trial	FBGs (30)	2.45 GHz-MW generator and 15 cm long antenna with an active tip of 31 mm (Microwave Ablation System, Surgnova Healthcare Technologies, Zhejiang)

**Table 5:** Studies related to temperature monitoring during HIFU ablation in bone.

Authors, Reference and Year	Type of Study	Type of Techniques	Type of Source
Geiger et al. [191], 2014	Clinical trial	MR-thermometry	ExAblate 2100 MR-guided focused ultrasound system (InSightec, Tirat Carmel, Israel)
Ramsay et al. [192], 2015	Clinical trial	MR-thermometry and 4 fiber optics	1.2 MHz-transducer
Lam et al. [193], 2016	Clinical trial	MR-thermometry	Not specified
Ozhinspky et al. [194], 2016	Ex vivo and in vivo animal trial	MR-thermometry and 3 fiber optics	Ultrasound system operating at 500 kHz (ExAblate 2100, InSightec, Israel)
Sharma et al. [195], 2017	Clinical trial	MR-thermometry	Ultrasound system Sonalleve V2 (Philips, Vantaa, Finland)
Guillemin et al. [196], 2019	Ex vivo animal trial	MR-thermometry and 1 fluoroptic sensor	Phased array HIFU transducer (Imasonic, Besançon, France)
Lena et al. [197]	Ex vivo and in vivo trials	MR-thermometry	HIFU Platform (Sonalleve MR-HIFU V2; Profound Medical, Mississauga, ON, Canada Mississauga, ON, Canada)

## Data Availability

Not applicable.

## Abbreviations

The following abbreviations are used in the manuscript:

OO	Osteoid osteoma
HT	Hyperthermia treatments
RFA	Radiofrequency ablation
LA	Laser ablation
MWA	Microwave ablation
CT	Computed tomography
MR	Magnetic resonance
HIFU	High intensity focused ultrasound
CEM	Cumulative equivalent minutes
FBG	Fiber Bragg grating sensors
emf	Electromotive force
PTC	Positive temperature coefficient
NTC	Negative temperature coefficient
HU	Hounsfield unit
PRF	Proton Resonance Frequency
TE	Echo time
RF	Radiofrequency
